# Regulation of Microglia Activity by Glaucocalyxin-A: Attenuation of Lipopolysaccharide-Stimulated Neuroinflammation through NF-κB and p38 MAPK Signaling Pathways

**DOI:** 10.1371/journal.pone.0055792

**Published:** 2013-02-05

**Authors:** Byung-Wook Kim, Sushruta Koppula, Seong-Su Hong, Sae-Bom Jeon, Ji-Hye Kwon, Bang-Yeon Hwang, Eun-Jung Park, Dong-Kug Choi

**Affiliations:** 1 Department of Biotechnology, College of Biomedical and Health Science, Konkuk University, Chungju, South Korea; 2 College of Pharmacy and Medical Research Center (CICT), Chungbuk National University, Cheongju, South Korea; 3 Branches of Immune and Cell Therapy, National Cancer Center, Goyang, South Korea; Virginia Commonwealth University, United States of America

## Abstract

Microglial cells are the resident macrophages and intrinsic arm of the central nervous system innate immune defense. Microglial cells become activated in response to injury, infection, environmental toxins, and other stimuli that threaten neuronal survival. Therefore, regulating microglial activation may have therapeutic benefits that lead to alleviating the progression of inflammatory-mediated neurodegeneration. In the present study, we investigated the effect of glaucocalyxin A (GLA) isolated from *Rabdosia japonica* on the production of pro-inflammatory mediators in lipopolysaccharide (LPS)-stimulated primary microglia and BV-2 cells. GLA significantly inhibited LPS-induced production of nitric oxide and reversed the morphological changes in primary microglia. Further, GLA suppressed expression of inducible nitric oxide synthase and cyclooxygenase-2 dose-dependently at the mRNA and protein levels. The production of proinflammatory cytokines such as tumor necrosis factor-α, interleukin-1β (IL)-1β, and IL-6 were inhibited by suppressing their transcriptional activity. Furthermore, GLA suppressed nuclear factor-κB activation by blocking degradation of IκB-α and inhibited the induction of lipocalin-2 expression in LPS-stimulated BV-2 cells. Mechanistic study revealed that the inhibitory effects of GLA were accompanied by blocking the p38 mitogen activated protein kinase signaling pathway in activated microglia. In conclusion, given that microglial activation contributes to the pathogenesis of neurodegenerative diseases, GLA could be developed as a potential therapeutic agent for treating microglia-mediated neuroinflammatory diseases.

## Introduction

Dysregulation of the neuroimmune system has been suggested to play a decisive role in the pathogenesis of several neurodegenerative disorders. Microglia, the resident immune cells of the central nervous system, become activated and induce significant and highly detrimental neurotoxic effects by excessively producing a large array of cytotoxic and proinflammatory factors [1], [2], [3]. Microglia enhance and amplify neuronal damage induced by pathological stimuli and toxins when they are over activated, which, in turn, induces more widespread damage to neighboring neurons through a process called reactive microgliosis [4], [5].

Therefore, the ideal therapeutic approach would involve early attenuation of the microglial response to levels that are no longer deleterious to the neuronal environment, and drugs targeting specific aspects of the microglia-related cascade may prove successful. Lipopolysaccharide (LPS) is a common toxin used to investigate the impact of inflammation on neuronal death, and microglial cells are necessary for LPS-induced neurotoxicity [6]. BV-2 microglial cells respond to the LPS endotoxin by synthesizing inflammatory factors such as nitric oxide (NO), cyclooxygenase-2 (COX-2), tumor necrosis factor-alpha (TNF-α), interleukin (IL)-1β, and IL-6. These microglial products are thought to be responsible for neuroglia-mediated neurotoxicity [7].

We isolated and purified glaucocalyxin A (GLA) from *Rabdosia japonica* (*R. japonica*) var. *galucocalyx* (Labiatae) as part of our systematic approach to drug discovery and identifying potential compounds that ameliorate the inflammatory-mediated events seen in neurodegenerative diseases. *R. japonica* is a perennial herb that is distributed widely in East Asia, and the entire *R. japonica* extract has been used traditionally as a folk medicine for treating gastrointestinal disorders, tumors, and inflammatory diseases [8], [9]. In this study, the anti-neuroinflammatory effect of GLA and the underlying mechanisms involved were investigated in LPS-stimulated microglial cells.

## Materials and Methods

### Reagents

LPS (*E. coli* 0111:B4), Tween-20, bovine serum albumin (BSA), dimethyl sulfoxide (DMSO), p-nitrophenyl phosphate, 3-(4, 5-dimethylthiazol-2-yl)-2, 5- phenyltetrazolium bromide (MTT), and pyrrolidine dithiocarbamate (PDTC) were purchased from Sigma-Aldrich (St. Louis, MO, USA). Dulbecco’s Modified Eagle Medium, fetal bovine serum, phosphate buffered saline (PBS), and other cell culture reagents were obtained from Gibco/Invitrogen (Grand Island, NY, USA). Protease inhibitor cocktail tablets and phosphatase inhibitor cocktail tablets were supplied by Roche (Roche, Indianapolis, IN, USA). COX-2, nuclear factor (NF)-κB p65, and nucleolin antibodies were obtained from Santa Cruz Biotechnology (Santa Cruz, CA, USA). Inducible nitric oxide synthase (iNOS), IκB-α, phospho (p)-IκB-α, p38, p-p38, and β-actin were supplied by Cell Signaling Technology (Danvers, MA, USA). The mouse lipocalin-2 (LCN2) antibody was purchased from R&D Systems (Minneapolis, MN, USA). Nuclear extract kit was purchased from Thermo Scientific (Rockford, IL, USA), prostaglandin E2 (PGE_2_) enzyme-linked immunoassay (EIA) kit was obtained from Cayman Chemical Co. (Ann Arbor, MI, USA), and nitric oxide synthase (NOS) 2 and inducible NOS (iNOS) 2 EIA kits were purchased from Uscn Life Science Inc. (Wuhan, China).

### Plant Material

The aerial parts of *R. japonica* were collected from the herb garden at Chungbuk National University, Cheongju, Korea. The plant material was authenticated by a taxonomist, and a voucher specimen (CBNU0909) was deposited at the herbarium of the College of Pharmacy, Chungbuk National University, Korea. The aerial parts of *R. japonica* were shade dried and extracted three times with methanol at room temperature with a starting material of 500 g. The solvent obtained was evaporated under reduced pressure, and the final yield (35 g) was freeze dried and stored in a refrigerator.

### Isolation of GLA using Bioactivity-guided Fractionation

**Figure 1:**
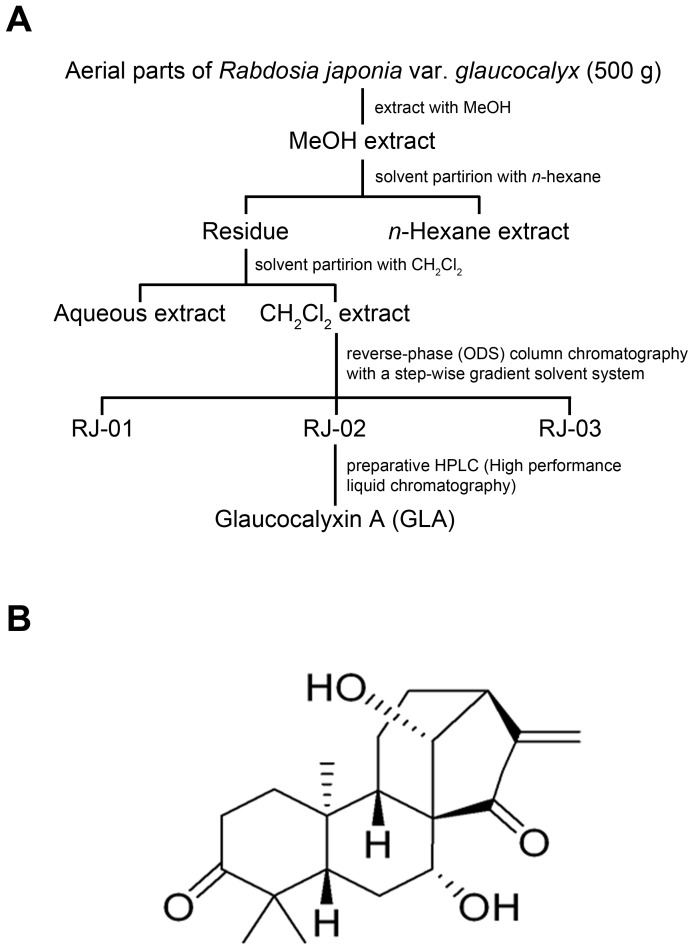
Isolation and identification of glaucocalyxin-A (GLA). A: Isolation procedure. B: Chemical structure of GLA.

The freeze dried extract of the *R. japonica* aerial parts was suspended in water and partitioned with n-hexane and dichloromethane (CH_2_Cl_2_), successively. The CH_2_Cl_2_ fraction was subjected to reverse-phase silica gel (ODS) column chromatography using, water (H_2_O) and acetonitrile (MeCN) gradient to produce three fractions named RJ-01, RJ-02, and RJ-03. The active subfraction RJ-02 was further separated by preparative high performance liquid chromatography (HPLC) [J’-sphere ODS H-80 150×20 mm; MeCN/H_2_O (4∶6); flow rate: 6.0 mL/min] to produce a white amorphous powder named GLA (16.2 mg, Fig. 1A). The electrospray ionization mass spectroscopy (EI-MS) spectrum was obtained on a VG Autospec Ultima mass spectrometer. ^1^H, ^13^C nuclear magnetic resonance (NMR), distortionless enhancement by polarization transfer, and heteronuclear multiband connectivity spectra were recorded using a Bruker DRX 500 NMR spectrometer. The GLA structure (Fig. 1B) was analyzed by NMR spectroscopy and was confirmed by comparison of its physicochemical and spectroscopic data as reported previously [10]. GLA: white amorphous powder; EI-MS *m/z*: 332 [M]^+^; ^1^H-NMR (500 MHz, pyridine-*d*_5_) δ: 6.31 (1H, s, H-17a), 5.38 (1H, s, H-17b), 5.06 (1H, br s, H-14), 4.71 (1H, dd, *J* = 11.5, 5.0 Hz, H-7), 3.24 (1H, br s, H-13), 2.51-2.43 (2H, m, H-2), 2.08 (1H, m, H-12α), 2.01 (1H, m, H-12β), 1.73 (1H, overlap, H-1α), 1.66 (1H, overlap, H-5), 1.63 (2H, m, H-6), 1.44 (1H, br s, H-9), 1.40 (2H, m, H-11), 1.20 (1H, m, H-1β), 1.07 (6H, s, Me-18, 19), 1.00 (3H, s, Me-20); ^13^C-NMR (125 MHz, pyridine-*d*_5_) δ: 215.9 (C-3), 207.6 (C-15), 149.6 (C-16), 116.5 (C-17), 75.3 (C-14), 73.5 (C-7), 61.6 (C-8), 53.3 (C-9), 51.5 (C-5), 46.8 (C-4), 46.7 (C-13), 38.9 (C-10), 38.3 (C-1), 34.0 (C-2), 31.1 (C-6), 30.8 (C-12), 27.4 (C-18), 21.1 (C-19), 18.4 (C-11), 18.1 (C-20).

### Cell Culture

Primary microglia was cultured from the cerebral cortices or substantia nigra of 1- to 3-day-old Sprague-Dawley rats. Briefly, tissues were triturated into single cells in MEM containing 10% FBS and were plated in 75-cm^2^ T-flasks (0.5 hemisphere/flask) for 2 weeks. The microglia were detached from the flasks by mild shaking and applied to a nylon mesh to remove astrocytes and cell clumps. Cells were plated in 6-well plates (5×10^5^ cells/well), 60-mm^2^ dishes (8×10^5^ cells/dish), or 100-mm^2^ dishes (2×10^6^ cells/dish). One hour later, the cells were washed to remove unattached cells before use in experiments. The purity of microglia cultures was assessed using CD-11b antibody and more than 90% of cells were stained positively.

The BV-2 cell (a mouse microglial cell line) was originally developed by Dr. V. Bocchini (University of Perugia, Perugia, Italy), and it was generously provided to us by Dr. K. Suk (Kyung-Pook National University, Daegu, Korea). The immortalized murine BV-2 cell line that exhibits both the phenotypic and functional properties of the reactive microglia cells [11] were grown and maintained in Dulbecco’s Modified Eagle’s Medium (DMEM) supplemented with 5% heat-inactivated FBS, 50 µg/mL of penicillin–streptomycin and maintained in a humidified incubator at 37°C, with 5% CO2. Primary microglial cells were seeded at a density of 5×10^5^ cells/mL and BV-2 cells at 5×10^4^ cells/mL. In separate experiments, primary microglia were pretreated for 1 h with various concentrations of GLA (0.1 and 0.5 µM) and BV-2 cells were treated with 0.1, 1, and 5 µM GLA before incubation in LPS-containing medium (25 ng/mL and 100 ng/mL respectively (*E. coli* 0111:B4, Sigma).

### NO Production Assay

NO production was assayed by measuring the levels of nitrite, the stable NO metabolite, in culture media. Nitrite accumulation in media was determined using a colorimetric assay with Griess reagent [12]. Cells (5×10^4^ cells/mL) were seeded in 96-well plates in 100 µL culture medium and stimulated with LPS (25 or 100 ng/mL) for 24 h. A 50 µL aliquot of culture supernatant was reacted with an equal volume of Griess reagent (0.1% naphthylethylenediamine and 1% sulfanilamide in 5% H_3_PO_4_) in 96-well plates for 10 min at room temperature in the dark. Nitrite concentrations were determined using standard solutions of sodium nitrite prepared in medium. Absorbance was determined at 540 nm using a microplate reader.

### iNOS Enzyme Activity Assay

To obtain the total cell lysate, 80 µL of RIPA buffer was added to the BV-2 cells (5×10^5^ cells/mL) cultured in 6-well plates. The cells were scraped, incubated for 10 min on ice and centrifuged at 14,000×rpm for 10 min at 4°C. The protein concentration was determined by the DC protein assay from Bio-Rad (Hercules, CA, USA), and 10 µg of whole cell lysate were used for iNOS activity. The iNOS enzyme activity in lysate were determined spectrophotometrically (Tecan Trading AG, Switzerland) at 450 nm. Each experiment was performed in triplicate.

### PGE_2_ Production

Culture supernatants were collected by centrifugation, and the PGE_2_ concentration was determined by enzyme immunoassay using a PGE_2_ EIA kit. The absorbance at 420 nm was determined using a microplate reader (Molecular Devices, Sunnyvale, CA, USA). Each experiment was performed in triplicate.

### Reverse Transcription-polymerase Chain Reaction (RT-PCR)

Total RNA was extracted using TRIZOL (Invitrogen, Carlsbad, CA, USA). RNA (2.5 µg) was reverse-transcribed using a Superscript™-III kit (Invitrogen), according to the manufacturer’s instruction. PCR amplification was carried out in a 50 µL PCR reaction mixture containing 10 mM Tris-HCl (pH 8.3), 50 mM KCl, 2 mM MgCl_2_, 20 pmol of each primer set, two units of Taq DNA polymerase (Bioneer Co., Daejeon, Korea), 0.2 mM dNTPs, and 2 µL of cDNA. The PCR conditions were described previously [13].

### Western Blot Analysis

Cells were lysed on ice in lysis buffer consisting of 50 mM Tris-HCl (pH 8.0), 150 mM NaCl, 1% Triton X-100, 0.5% sodium deoxycholate, 0.1% sodium dodecyl sulfate (SDS), 1 mM EDTA (Sigma), 1% protease inhibitor cocktail, and 1% phosphatase inhibitor cocktail (Roche). After centrifugation, the protein concentration of the supernatant was assayed using a Bio-Rad DC Protein Assay kit. Lysate samples containing 20 or 40 µg of protein were fractionated by SDS-10% polyacrylamide gel electrophoresis and then electroblotted onto polyvinylidene difluoride membranes (Millipore, Bedford, MA, USA). The membranes were probed with primary antibodies to anti-iNOS, anti-IκB-α, anti-phospho-IκB-α (1∶1000), anti-β-actin (1∶2000) anti-COX-2, anti p65 (1∶1000), and anti nucleolin (1∶500). The blots were visualized by a PowerOpti-ECL (Animal Genetics Inc, Tallahassee, FL, USA) detection system according to the manufacturer’s procedure and the antibody-specific bands were scanned by a Luminescent Analyzer, LAS-3000 and quantified using Fuji Multigage software V3.1 (Fuji, Tokyo).

### Double-immunofluorescence Labeling Assay

Primary microglia were plated onto round coverslips (Fisher Scientific) and fixed with ice-cold 100% methanol. Fixed microglia were permeabilized with PBS containing 0.1% Triton X-100 (PBS-T) and blocked with 1% BSA (Sigma-Aldrich). The product was then washed with PBS-T and incubated overnight at 4°C with OX-42 (1/100) antibody. Samples were rinsed three times for 5 min with PBS-T. Each coverslip was mounted on slides with mounting solution, and the cells were observed with a Carl Zeiss LSM510 confocal microscope (Carl Zeiss, Oberkochen, Germany).

BV-2 microglia cells were cultured on sterile cover slips in 24-well plates and treated with compounds and LPS to detect the intracellular location of the NF-κB p65 subunit. At 60 min after the LPS treatment, the cells were fixed with methanol for 20 min at -20°C and washed with PBS for 5 min. The fixed cells were then permeabilized with 0.5% Triton X-100 in PBS for 1 h at room temperature, washed with 0.05% Tween-20 in PBS for 10 min, followed by 0.05% Tween-20/1% BSA in PBS for 5 min. The permeabilized cells were then treated with 1 mg/ml monoclonal mouse anti-human NF-κB (p65) for 60 min at room temperature and washed with 0.05% Tween-20/1% BSA in PBS for 5 min. The cells were then incubated in a 1∶2000 dilution of Alexa Fluor 568-labeled goat anti-mouse antibody (Invitrogen) for 60 min at room temperature and washed with 0.05% Tween-20 in PBS for 5 min, followed by PBS for 5 min. The cells were then stained with 0.1 µM of Hoechst (Invitrogen) staining solution for 20 min at 37°C and then washed. Finally, all images were captured with a Carl Zeiss Axio40 fluorescence microscope.

### Statistical Analyses

Data are presented as means ± standard errors of at least three separate experiments conducted in triplicate. Comparisons between groups were analyzed using One-Way analysis of variance (ANOVA) followed by *Bonferroni post hoc* multiple comparisons test with using software Graphpad Prism V5.01. (GraphPad Software Inc., San Diego, CA, USA). P-values less than 0.05 were considered to be statistically significant.

## Results

### Inhibition of NO Production by GLA in LPS-stimulated Primary Microglial and BV-2 Cells

**Figure 2:**
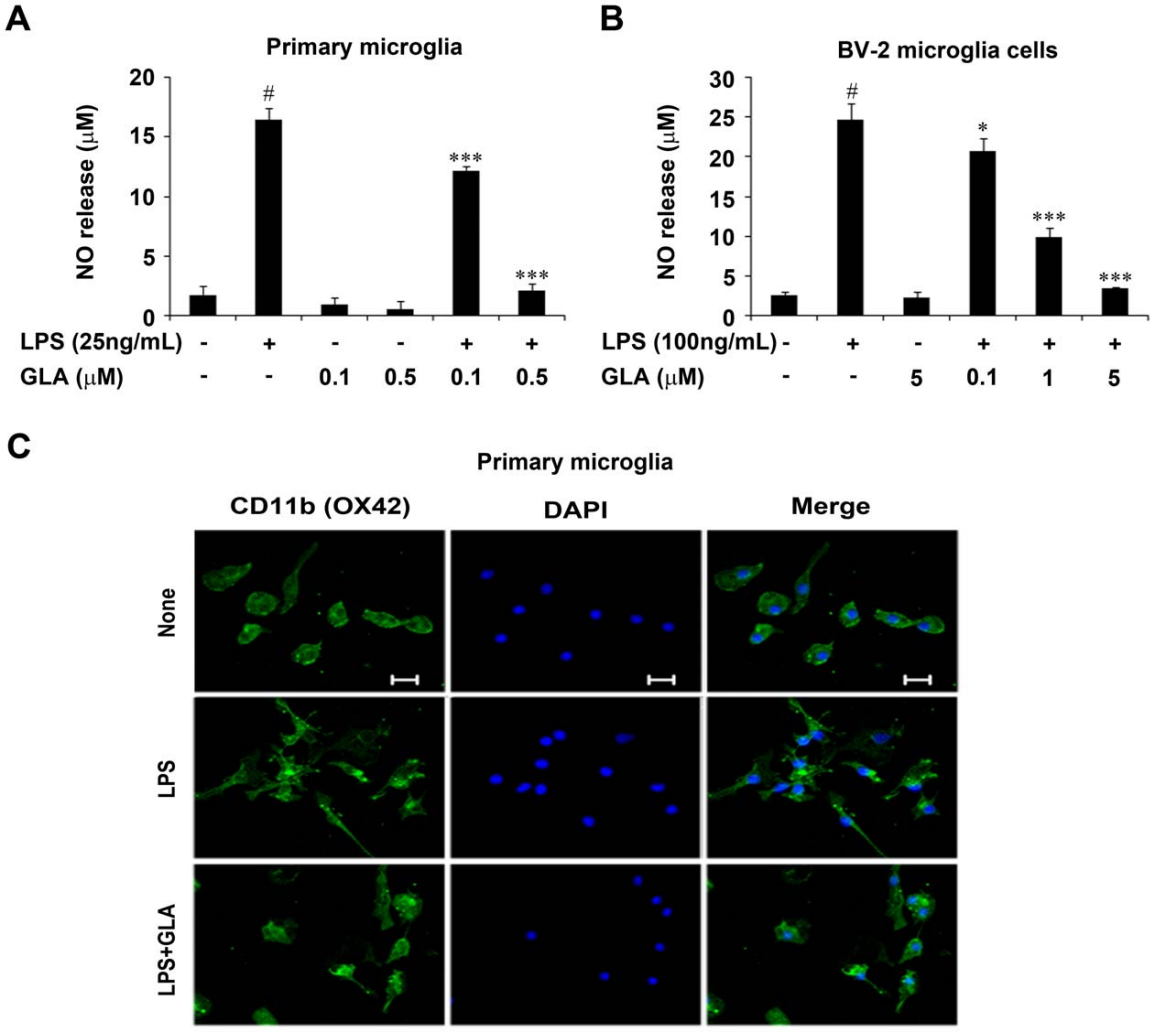
Effect of glaucocalyxin-A (GLA) on nitric oxide (NO) production in lipopolysaccharide (LPS)-stimulated microglial cells. A: Rat primary microglial cells were pre-treated with the indicated concentrations (0.1 µM or 0.5 µM) of GLA for 1 h before incubating with LPS (25 ng/mL) for 24 h. Nitrite levels were measured in culture media by the Griess reaction. B: BV-2 microglia cells were pre-treated with the indicated concentrations of GLA (0.1, 1, or 5 µM) for 1 h before stimulating with LPS (100 ng/mL) for 24 h. C: Primary microglial cells were treated with GLA (0.1 µM) and LPS(25 ng/ml) for 18 h. Cells were stained with CD11b (OX-42) antibody and changes in morphology was observed using confocal microscopy (scale bar, 20 µm). Data are mean ± S.E.M. (n = 3) for three independent experiments. ^#^P<0.001, compared with control group; *P<0.05, **P<0.01 and ***P<0.001 compared with LPS-treated group by One-Way analysis of variance (ANOVA) followed by *Bonferroni’s* multiple comparison test.

To evaluate the effect of GLA on LPS-induced NO production, primary microglial cells were pre-treated for 1 h with different concentrations of GLA (0.1 or 0.5 µM), followed by LPS (25 ng/mL) for 24 h, and the levels of NO in culture media were determined using the Griess assay. As shown in Fig. 2A, when primary microglial cells were stimulated with LPS (25 ng/mL) for 24 h, the levels of nitrite, a stable oxidized product of NO, increased markedly in the culture medium to 16.39±0.55 µM compared with the control at 1.72±0.39 µM. When pre-treated with GLA at different doses, the LPS-induced increase in NO production decreased to 12.19±0.19 µM and 2.09±0.33 µM respectively, in a concentration-dependent manner. Furthermore, we investigated whether GLA had similar inhibitory effects in BV-2 cells. As shown in Fig. 2B, LPS treatment (100 ng/mL) alone markedly induced NO production (24.61±0.82 µM) in BV-2 cells compared to that in the control (2.55±0.41 µM). Pre-treatment with GLA (0.1, 1, or 5 µM) significantly suppressed the increase in NO production to 20.77±0.65, 9.96±0.45, and 3.49±0.06 µM respectively. The morphological changes of normal cultured primary microglia observed using confocal microscopy showed round or amoeboid shape, whereas LPS (25 ng/mL)-stimulated primary microglia exhibited marked changes from a round or amoeboid shape to a multipolar rod or ramified morphology. Pretreatment with GLA at 0.1 µM reversed this altered changes in morphology (Fig. 2C).

Our results indicate that GLA has a potent effect for inhibiting increased NO production in LPS-stimulated primary microglia and BV-2 cells. GLA treatment alone at the indicated concentrations did not alter overall production of NO in primary microglia or BV-2 cells.

### GLA Attenuates LPS-stimulated Increase in iNOS Levels and Enzyme Activity in Microglial Cells

**Figure 3:**
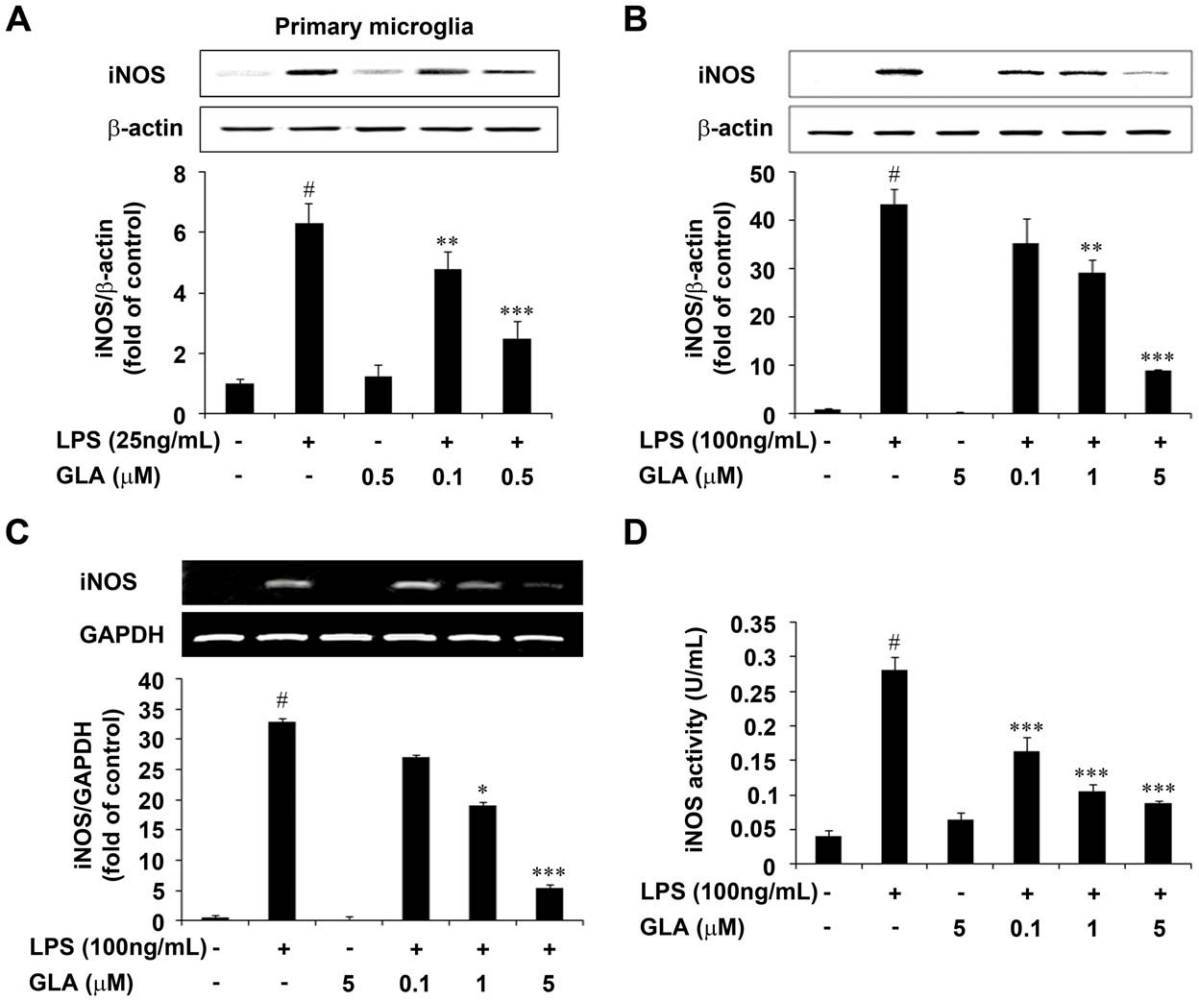
Effect of glaucocalyxin-A (GLA) on inducible nitric oxide synthase (iNOS) expression and enzyme activity in lipopolysaccharide (LPS)-stimulated microglia. Cells were pre-treated with the indicated concentrations of GLA for 1 h before incubating with LPS (25 ng/mL or 100 ng/mL) for 18 h A: Primary microglia, B: BV-2 microglia. Lysates were analyzed by immunoblotting with an anti-iNOS antibody. Representative quantification data were shown in the lower panel. Results are expressed as a ratio of iNOS to β-actin. C: BV-2 cells were pre-treated with the indicated concentrations of GLA for 1 h before incubating with LPS (100 ng/mL) for 6 h. The total RNA was isolated and iNOS mRNA level was determined by RT-PCR. Quantification data are shown in the lower panel. Results are expressed as a ratio of iNOS to GAPDH. D: BV-2 microglial cells were pre-treated with the indicated concentrations of GLA for 1 h before incubating with LPS (100 ng/mL) for 18 h. iNOS activity was analyzed with enzyme immunoassay kit. Data are mean ± S.E.M. (n = 3) for three independent experiments. ^#^P<0.001 compared with control group; *P<0.05, **P<0.01 and ***P<0.001 compared with LPS-treated group by One-Way analysis of variance (ANOVA) followed by *Bonferroni’s* multiple comparison test.

As shown in Fig. 3A, primary microglia showed increased protein levels when stimulated with LPS (25 ng/mL). Pre-treatment with GLA at indicated concentrations (0.1 and 0.5 µM) attenuated this increase significantly. Further BV-2 cells stimulated with LPS resulted in increased iNOS expression and subsequent iNOS enzyme production. Pre-treatment with GLA at various concentrations (0.1, 1, or 5 µM) significantly inhibited iNOS mRNA and protein levels (Fig. 3B and 3C), compared with those in the LPS-treated control. In addition, GLA reduced LPS-stimulated iNOS enzyme activity in BV-2 cells significantly (p<0.001). The data showed a significant reduction (68.71±5.36%) in enzyme activity following GLA treatment (5 µM) in LPS-treated BV-2 cells (Fig. 3C).

### Attenuation of COX-2, PGE_2_ and Proinflammatory Cytokine Levels by GLA in LPS-Stimulated Microglial Cells

**Figure 4:**
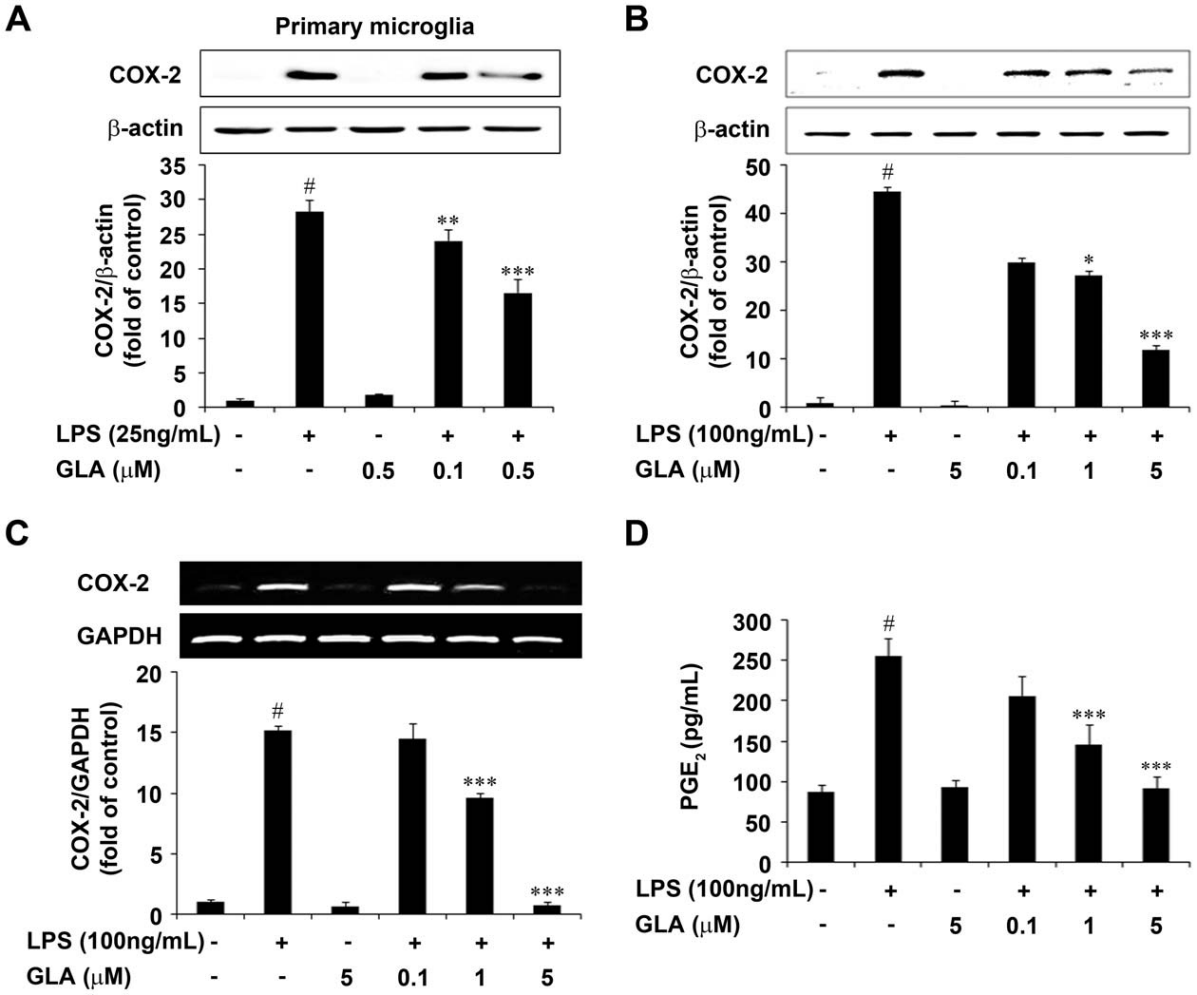
Effect of glaucocalyxin-A (GLA) on cyclooxygenase-2 (COX-2) and prostaglandin E_2_ (PGE_2_) expression in lipopolysaccharide (LPS)-stimulated microglia. Cells were pre-treated with the indicated concentrations of GLA for 1 h before incubating with LPS (25 ng/mL or 100 ng/mL) for 18 h A: Primary Microglia, B: BV-2 cells. Results are expressed as a ratio of COX-2 to β-actin. Representative quantification data was shown in the lower panel. C: Cells were pre-treated with the indicated concentrations of GLA for 1 h and then stimulated with LPS (100 ng/mL) for 6 h. Total RNA was prepared and COX-2 mRNA level was determined by RT-PCR. Results are expressed as the ratio of COX-2 to GAPDH. Quantification data are shown in the lower panel. D: PGE_2_ levels were analyzed with an enzyme immunoassay kit. Absorbance was measured at 420 nm spectrophotometrically. Data are mean ± S.E.M. (n = 3) for three independent experiments. ^#^P<0.001 compared with control group; *P<0.05 and ***P<0.001 compared with LPS-treated group by One-Way analysis of variance (ANOVA) followed by *Bonferroni’s* multiple comparison test.

LPS stimulation to primary microglia led to a significant increase in COX-2 protein levels when compared to controls (p<0.001). Pre-treatment with GLA at indicated concentrations (0.1 and 0.5 µM) inhibited the LPS-stimulated increase in COX-2 levels (p<0.01 and p<0.001, respectively) significantly (Fig. 4A). In LPS-stimulated BV-2 cells, GLA significantly attenuated both protein level and mRNA expression in a concentration dependent manner (Fig. 4B and 4C). In addition, treatment of BV-2 cells with LPS for 18 h, caused nearly a 2.7-fold increase in PGE_2_ release when compared with untreated controls. However, pre-treatment with GLA inhibited the LPS-stimulated increase in the production of PGE_2_ in a dose-dependent fashion (Fig. 4D).

**Figure 5:**
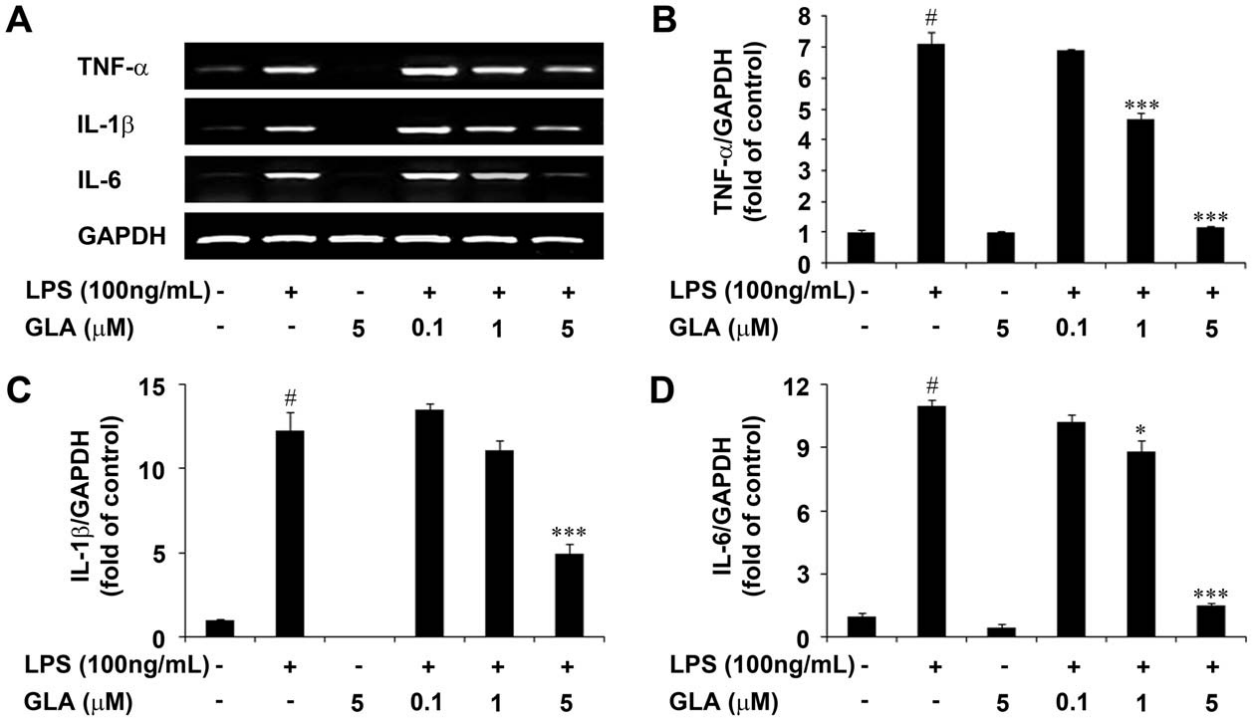
Effect of glaucocalyxin-A (GLA) on pro-inflammatory cytokines such as tumor necrosis factor-α (TNF-α), interleukin-1β (IL-1β), and IL-6 in lipopolysaccharide (LPS)-stimulated BV-2 microglia. A: Cells were pre-treated with the indicated doses of GLA for 1 h before LPS (100 ng/mL) treatment. The levels of TNF-α, IL-1β, and IL-6 mRNA were determined by RT-PCR. Representative densitometry analysis of B: TNF-α, C: IL-1β, and D: IL-6 compared with GAPDH mRNA, respectively. Data are mean ± S.E.M. (n = 3) for three independent experiments. ^#^P<0.001 compared with the control group; **P<0.01 and ***P<0.001 compared with the LPS-treated group by One-Way analysis of variance (ANOVA) followed by *Bonferroni’s* multiple comparison test.

Pro-inflammatory cytokines such as TNF-α, IL-1β, and IL-6 play central roles in microglia-mediated inflammation. Therefore, the effects of GLA on proinflammatory cytokine production in LPS-stimulated BV-2 microglial cells were evaluated. BV-2 cells were incubated with GLA (0.1, 1.0, or 5.0 µM) in the presence or absence of LPS (100 ng/mL). TNF-α, IL-1β, and IL-6 were not expressed at detectable levels under normal culture conditions, but the expression of these cytokines was significantly upregulated after 6 h of treatment with LPS (100 ng/mL). Pre-treatment with GLA significantly inhibited LPS-induced TNF-α, IL-1β, and IL-6 production, compared with that in the LPS-treated controls (Fig. 5A). The representative quantification data revealed that LPS-stimulated proinflammatory cytokine mRNA levels (TNF-α, IL-1β and IL-6) were significantly decreased following GLA treatment (Fig. 5B, 5C and 5D, respectively), suggesting that GLA negatively regulates TNF-α, IL-1β, and IL-6 production.

### Effect of GLA on LPS-induced NF-κB Nuclear Translocation and IκB-α Degradation in Microglial Cells

**Figure 6:**
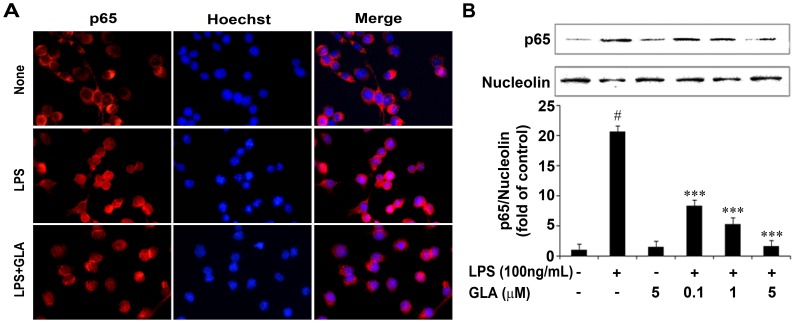
Inhibitory effect of glaucocalyxin-A (GLA) on nuclear factor (NF)-κB activity in lipopolysaccharide (LPS)-stimulated BV-2 microglia. A: BV-2 microglia cells were seeded at a density of 5×10^4^cells/well on 24-well plates. Cells were stimulated with LPS (100 ng/mL) in the absence or presence of GLA (5 µM) added 1 h before stimulation. At 30 min after adding the LPS, the sub-cellular location of the NF-κB p65 subunit was determined by immunofluorescence assay. B: Cells were treated with the indicated dose of GLA 30 min before LPS (100 ng/mL) treatment. Total nuclear protein was subjected to 10% sodium dodecyl sulfate-polyacrylamide gel electrophoresis (SDS-PAGE) followed by Western blotting using anti-NF-κB p65. Densitometry analysis of NF-κB p65 is shown in the lower panel. Results are expressed as a ratio of NF-κB p65 to nucleolin. Data are mean ± S.E.M. (n = 3) for three independent experiments. ^#^ P<0.001 compared with control group; *P<0.05, **P<0.01, and ***P<0.001 compared with LPS-treated group by One-Way analysis of variance (ANOVA) followed by *Bonferroni’s* multiple comparison test.

**Figure 7:**
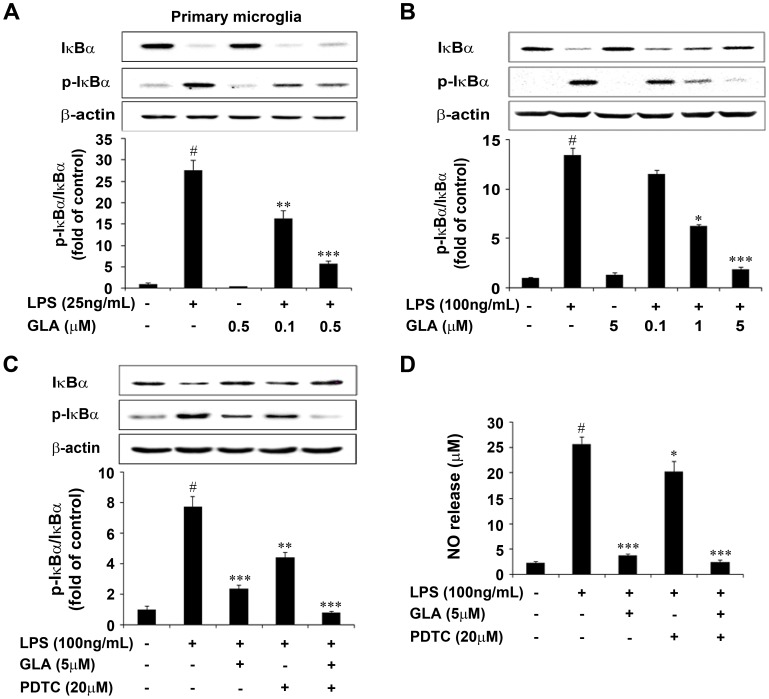
Inhibitory effect of glaucocalyxin-A (GLA) on IκB-α phosphorylation and degradation in lipopolysaccharide (LPS)-stimulated microglia cells. Cells were treated with the indicated dose of GLA for 1 h before LPS (100 ng/mL) treatment for 30 min. A: Primary Microglia, B: BV-2 cells C: BV-2 cells were pre-treated with 20 µM PDTC for 2 h before 5 µM GLA was added for 1 h and before incubating with LPS (100 ng/mL) for 30 min. Lysates were analyzed by immunoblotting with an anti p-IκB-α/IκB-α antibody. Representative densitometric analyses was shown in the lower panels. Results are expressed as a ratio of p-IκB-α to IκB-α. D: BV-2 microglial cells were pre-treated with 20 µM PDTC for 2 h before 5 µM GLA was added and 1 h before incubating with LPS (100 ng/mL) for 24 h. Data are mean ± S.E.M. (n = 3) for three independent experiments. ^#^P<0.001 compared with control group; *P<0.05, **P<0.01, and ***P<0.001 compared with LPS-treated group by One-Way analysis of variance (ANOVA) followed by *Bonferroni’s* multiple comparison test.

Mounting evidence suggests that increased production of pro-inflammatory cytokines by LPS stimulation was mediated by the activation of NF-κB. Reports also revealed that NF-κB activation includes degradation of IκB-α through phosphorylation and subsequent nuclear translocation of p65 subunit of NF-κB [13], [14], [15], [16]. NF-κB regulates and plays an essential role in LPS-induced expressional levels of iNOS, COX-2 and various proinflammatory cytokines [17], [18]. Therefore, to further elucidate the mechanism of GLA, NF-κB (p65) transcription factor was examined in LPS-stimulated BV-2 microglial cells. As shown in Fig. 6A, immunofluorescence assay revealed that GLA might regulate the LPS-stimulated NF-κB activation. LPS-stimulated BV-2 cells were pre-treated with GLA for 30 min and NF-κB activity was evaluated by nuclear translocation of the p65 subunit of NF-κB (Fig. 6B). Further, the phosphorylation of IκB-α was also inhibited by GLA treatment in both primary microglia and BV-2 cells significantly (Fig. 7A and 7B). In addition, the effect of GLA-inhibited IκB-α phosphorylation was consistent and showed synergistic action when co-treated with specific NF-κB inhibitor, PDTC on LPS-stimulated BV-2 microglial cells (p<0.001, Fig. 7C). LPS-induced NO production also decreased prominently when GLA was co-treated with PDTC (p<0.001, Fig. 7D). These results indicate that GLA suppressed the production of NO, iNOS, COX-2 and pro-inflammatory cytokines (TNF-α, IL-1β, and IL-6) and this inhibition might be through regulation of NF-*κ*B activation and degradation of I*κ*B-*α* in LPS-stimulated BV-2 cells.

### GLA Inhibits p38 Mitogen-activated Protein Kinase (MAPK) Phosphorylation in LPS-Stimulated Microglia Cells

**Figure 8:**
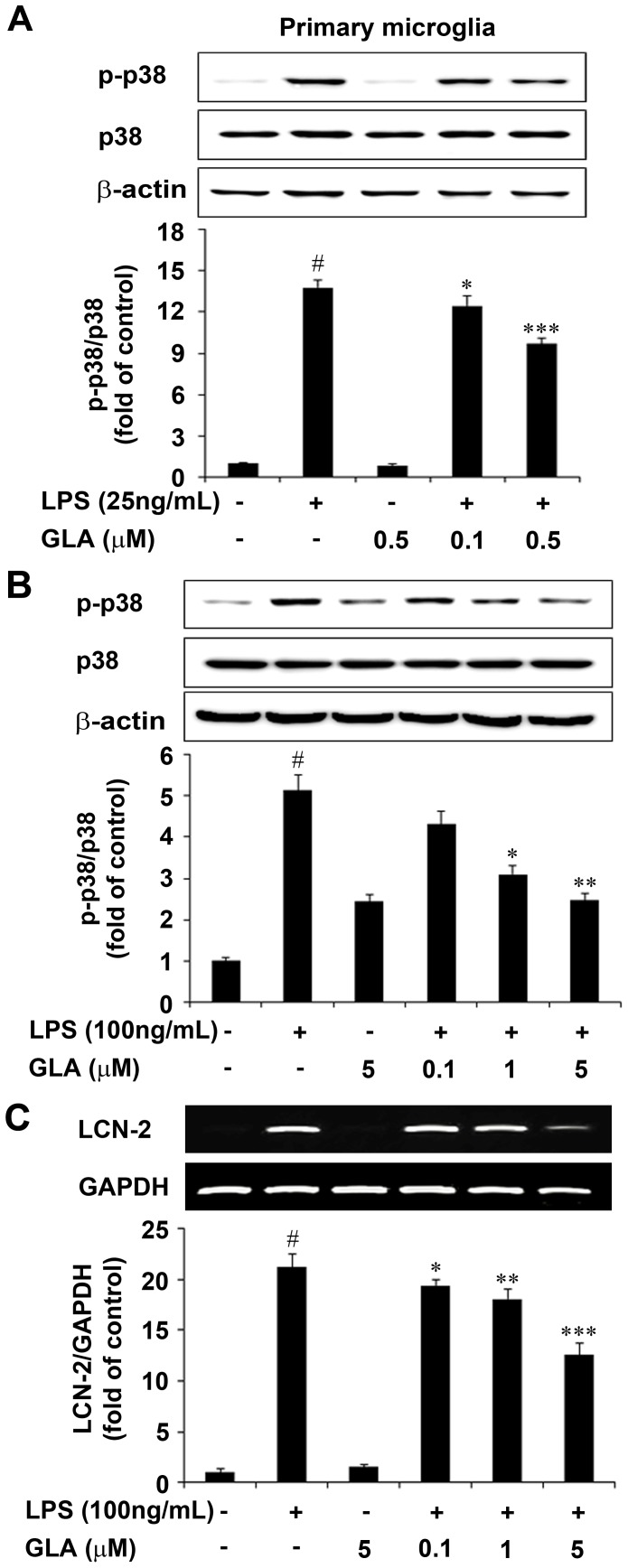
Inhibitory effects of glaucocalyxin-A (GLA) on phosphorylation of p38 mitogen activated protein kinase (MAPK) and expression of lipocalin-2 (LCN-2) in lipopolysaccharide (LPS)-stimulated microglia cells. Cells were pre-treated with indicated doses of GLA for 1 h before LPS treatment (100 ng/mL) for 30 min. A: Primary Microglia, B: BV-2 microglial cells. Total protein was subjected to 10% sodium dodecyl sulfate-polyacrylamide gel electrophoresis (SDS-PAGE) followed by Western blotting using anti-p38 MAPKs. Representative densitometric analysis of the p38 bands were shown in the lower panel. Results are expressed as a ratio of p-p38 to total p38. C: Cells were pre-treated with the indicated doses of GLA for 1 h before LPS (100 ng/mL) treatment. LCN-2 mRNA levels were determined by RT-PCR. GAPDH was used as an internal control for the RT-PCR analysis. Representative quantification data was shown in the lower panel. Results are expressed as a ratio of LCN-2 to GAPDH. Data are mean ± S.E.M. (n = 3) for three independent experiments. ^#^P<0.001 compared with control group; *P<0.05, **P<0.01, and ***P<0.001 compared with LPS-treated group by One-Way analysis of variance (ANOVA) followed by *Bonferroni’s* multiple comparison test.

The p38 mitogen activated protein kinase (p38 MAPK) plays a central role in the inflammatory cytokine response to immune challenge and consequently the development of sickness behavior [19]. To assess activation of this signaling kinase the levels of phosphorylated p38 MAPKs were determined in LPS-stimulated primary microglia and BV-2 cells. Pretreatment with GLA at indicated concentrations significantly inhibited LPS-stimulated p38 phosphorylation in primary microglial cells (p<0.05 and p<0.00, Fig. 8A). Consistent with primary microglial data GLA pre-treatment (0.5, 1.0 and 5.0 µM) also significantly inhibited p38 MAPK phosphorylation in BV-2 cells (Fig. 8B).

### GLA Suppresses LPS-induced Lipocalin-2 (LCN-2) Expression in BV-2 Cells

LCN-2 plays an important role as a mediator of microglial activation in response to diverse stimuli, including LPS [20], [21]. Recent reports have revealed that LCN-2 is induced by oxidative stress [22], and that it sequesters iron to protect the host from bacterial infection [23], [24]. LCN-2 suppresses cytokine production induced by LPS in macrophages [25] and regulates microglia activation and death in an autocrine manner [26]. We examined whether GLA could influence LCN-2 activation by RT-PCR analysis. As a result, LPS increased LCN-2 mRNA levels and GLA reduced LPS-induced LCN-2 mRNA expression (Fig. 8C).

## Discussion

In the present investigation, GLA showed a strong inhibition of microglia mediated deleterious consequences and possess anti-neuroinflammatory effects in LPS-stimulated primary microglia and BV-2 cells in several aspects. GLA inhibited NO production and restored the morphological changes observed in LPS-stimulated primary microglia. Furthermore, GLA inhibited LPS-induced NO and PGE_2_ production by suppressing iNOS and COX-2 mRNA and protein expression in BV-2 cells. The inhibitory effects of GLA might be regulated via NF-κB activation, translocation of NF-κB from the cytoplasm to the nucleus, and inhibiting LCN-2 expression, which were accompanied by blocking of the p38 MAPK signaling pathway in activated microglia.

Microglia-mediated neuroinflammation tends to be progressive and is the major component enhancing ongoing neurodegeneration. Overactivation and dysregulation of microglia via infection or injury can result in disastrous neurotoxic consequences such as release of proinflammatory cytokines, free radicals, and eicosanoids. These factors are believed to contribute to microglia-mediated neurodegeneration [27], [28], [29]. Although endogenous protective regulatory signals such as neuropeptides [6], [30], [31], cannabinoids [32], [33], [34], and anti-inflammatory cytokines (IL-1β and transforming growth factor-β) [35] in the brain inhibit microglial overactivation, the excessive inflammatory response might result in microglia initiating neuronal death and drive the progressive nature of neurodegenerative disease. Therefore, regulation of microglial activation could reduce neuroinflammation and further neuronal cell damage.

Activation of microglia is pivotal in the initiation of neuroinflammation. Prolonged activation of microglial cells leads to excessive release of NO by iNOS in the brain. NO, an important regulatory mediator involved in cell survival and death exerts a number of pro-inflammatory effects during several physiological and pathological processes. This inflammatory response is believed to be associated with several progressive neurodegenerative diseases [36]. COX-2 is upregulated in response to various inflammatory stimuli including overproduction of PGE_2_. Upregulation of COX-2 contributes to the development of many chronic inflammatory diseases [37], [38]. Therefore, agents that inhibit the release of NO and attenuate iNOS and COX-2 expression could be beneficial for preventing and delaying the progression of neuroinflammatory disease. In the present study, GLA treatment to primary microglia and BV-2 cells stimulated by LPS effectively decreased iNOS and COX-2 levels and the release of their respective end-products, NO and PGE_2_. This inhibition exhibited by GLA may be attributed to suppression of iNOS and COX-2 mRNA transcription and a subsequent reduction in protein expression. Furthermore, GLA exhibited significant inhibitory effects on NO production and restored the morphological changes observed in LPS-stimulated primary microglia.

The transcription factor NF-κB is a pleiotropic regulator of diverse genes involved in the immune and inflammatory responses. NF-κB activates several cellular signal transduction pathways that are involved in the production of iNOS, COX-2, and various cytokines [39], [40], [41]. The promoter region of the murine gene encoding iNOS and COX-2 contains NF-κB binding motifs. Activation of NF-κB results in phosphorylation, ubiquitination, and proteasome-mediated degradation of IκB proteins, followed by nuclear translocation. LPS has also been reported to activate NF-κB in microglia. As the expression of these proinflammatory mediators is modulated by NF-κB, blocking NF-κB transcriptional activity may be an important target for treating inflammatory diseases. Our findings show that GLA treatment blocked the degradation of IκB and of NF-κB nuclear translocation in BV-2 microglia stimulated by LPS, suggesting that the effects of GLA on the production of inflammatory mediators and cytokines are at least partially mediated by suppression of NF-κB signaling. Moreover, the inhibition of NF-κB transcriptional activity by GLA was consistent with the effect of the specific NF-κB inhibitor PDTC [42] when examined simultaneously in LPS-stimulated BV-2 microglial cells.

LCN-2 has diverse cellular functions such as iron delivery [23], [24], regulation of cell differentiation and migration [21], [43], cell death/survival [44], [45], and a role in inflammatory processes [46]. A recent study showed that LCN-2 is involved in the regulation of inflammation and cancer [47]. Thus, suppressing LCN-2 expression could modulate neuroinflammation. LCN-2 is strongly induced by the pro-inflammatory cytokine IL-1β via NF-κB activation [47], [48]. Increased levels of LCN2 are suggested to be a marker of inflammation. Therefore inhibiting NF-κB activation might regulate the induction of LCN-2 expression and help mitigate inflammatory processes. Data from our study revealed that GLA suppressed LCN-2 expression in LPS-stimulated BV-2 cells.

MAPKs are also involved in LPS-induced COX-2 and iNOS production via control of NF-κB activation in microglial cells [49], [50]. One of the important MAPK families, p38, is positively related to LPS signaling in microglial cells, which respond to pro-inflammatory cytokines [19], [51]. Moreover, p38 MAPK is a key mediator of cellular stressors such as inflammation and apoptosis. Both *in vitro* and *in vivo* studies have shown that p38 MAPK regulates the production of the pro-inflammatory cytokines, NO, and PGE_2_ by increasing cytokine release or messenger RNA transcription [52], [53]. Earlier reports revealed that inhibitors of p38 MAPK (SB203580) reduce LPS-induced iNOS protein levels [50], [54]. Results from our study indicate that GLA-mediated inhibition of p38 MAPK activation may also be a possible mechanism underlying its inhibitory action on iNOS, COX-2, and pro-inflammatory cytokines expression as well as NO production in LPS-stimulated BV-2 cells.

In conclusion, GLA showed significant anti-neuroinflammatory actions in microglial cells regulating via NF-κB and p38 MAPK Signaling Pathways. Given that microglial activation and the consequent release of various inflammatory components contributes to neurodegeneration, GLA could be developed as a potential therapeutic agent for treating microglia-mediated neuroinflammatory diseases.
